# Supplementary figures and images for: Haploinsufficiency of the essential gene Rps12 causes defects in erythropoiesis and hematopoietic stem cell maintenance
Source: eLife. 2023 Jun 5;12:e69322. doi: 10.7554/eLife.69322 (PMC10287158; doi:10.7554/eLife.69322)

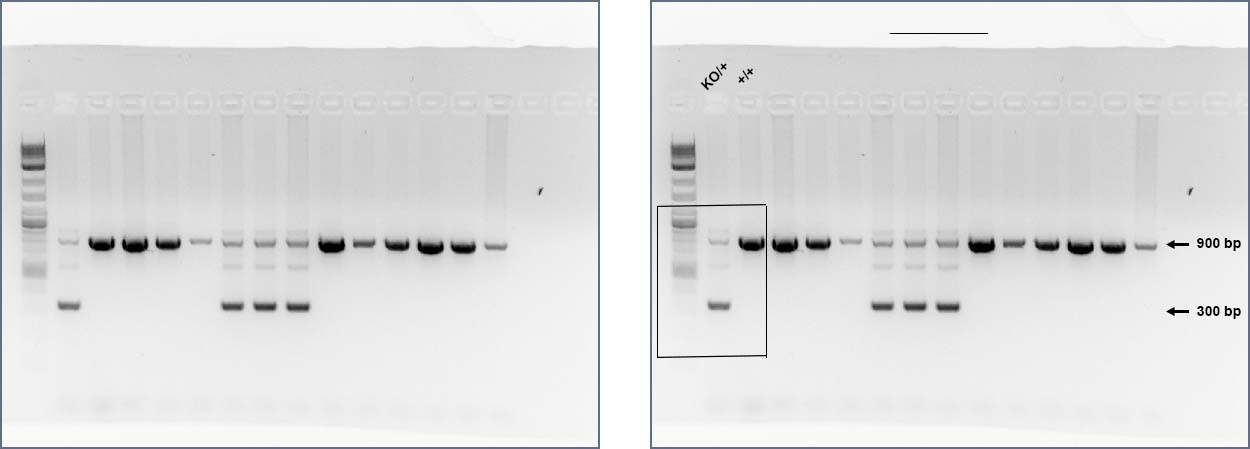

Supplement: Figure 1—figure supplement 1—source data 1. — The complete blot from which Figure 1—figure supplement 1B was derived is shown on the left. The 900 bp and 300 bp bands are marked on the right, as is the portion cropped for Figure 1—figure supplement 1B. [file elife-69322-fig1-figsupp1-data1.zip › Figure 1 Source data file 1.jpg]

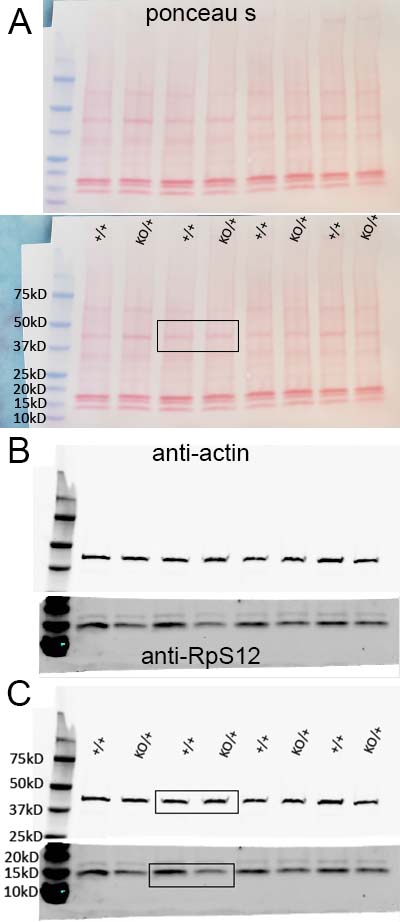

Supplement: Figure 3—figure supplement 2—source data 1. — (A) Complete blot corresponding to Figure 3—figure supplement 2C labeled with Ponceau S. The labeled blot is shown below including the region cropped for Figure 3—figure supplement 2C. (B) Complete blot corresponding to Figure 3—figure supplement 2C after western blotting with actin- and Rps12-specific antibodies (blot was cut in two). (C) Blots from panel B with labels, including regions cropped for Figure 3—figure supplement 2C. [file elife-69322-fig3-figsupp2-data1.zip › F3S2source230313.jpg]

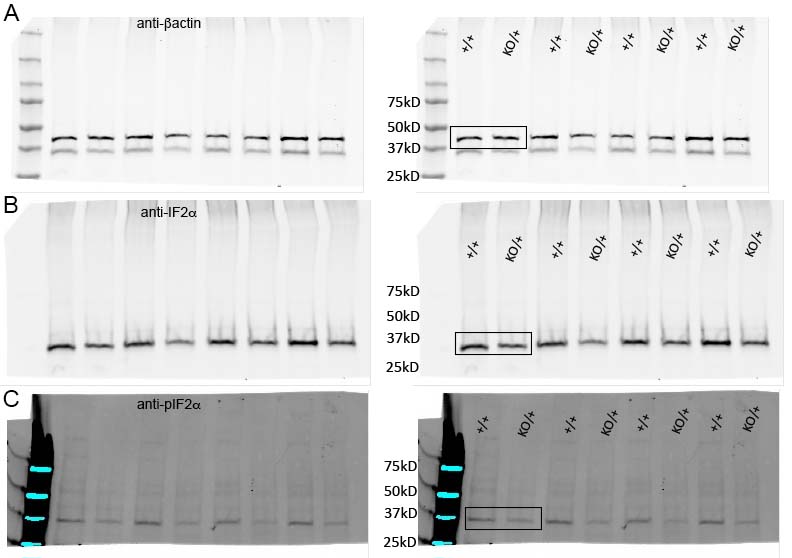

Supplement: Figure 7—source data 1. — (A) Complete blot shown in Figure 7I, after blotting with anti-βactin. On the right the blot is shown with labels and with the section cropped for Figure 7I indicated. (B) Complete blot showed after blotting with anti-IF2α. On the right the blot is shown with labels and with the section cropped for Figure 7I indicated. (C) Complete blot showed after blotting with anti-pIF2α. On the right the blot is shown with labels and with the section cropped for Figure 7I indicated. [file elife-69322-fig7-data1.zip › F7I source 230314.jpg]
